# Astrocyte-to-neuron conversion induced by spinal cord injury

**DOI:** 10.18632/oncotarget.13780

**Published:** 2016-12-02

**Authors:** Harun Najib Noristani, Florence Evelyne Perrin

**Affiliations:** University of Montpellier, Montpellier France; INSERM, Place Eugène Bataillon, Montpellier, Cedex, France

**Keywords:** spinal cord injury, astrocyte, transdifferentiation, neuron

Spinal cord injury (SCI) triggers pronounced astrocyte reactivity (astrogliosis) including astroglial proliferation and migration toward the injury site participating to the formation of a glial scar. Since the mid-20^th^ century, SCI-induced astrogliosis was mainly regarded as detrimental for successful axonal regeneration. However, more recent studies have shown astrogliosis as a multifactorial phenomenon involving specific morphological, molecular and functional alterations in astrocytes that can also exert beneficial effects [1, 2]. It was suggested, although not proven, that SCI-induced astrogliosis depends on multiple factors such as time after lesion, injury severity and distance to the lesion site. In a recent study we had attempted to uncover the molecular involvement of astrocytes after SCI by studying their transcriptomic alterations at different stages after moderate and severe lesions [3].

Aldehyde dehydrogenase 1 family member L1 (Aldh1l1) is a pan-astrocytic marker, hence using the Aldh1l1-EGFP transgenic mice, combined with fluorescence-activated cell sorting (FACS), we isolated pure astrocyte population at different stages following SCI. Choosing lateral hemisection and complete section of the spinal cord, as moderate and severe injury models, we investigated astrocytic response at 1 and 2 weeks after lesion. We subsequently carried out astrocyte-specific RNA-sequencing and pathway analyses to unveil the molecular signature of injuries-induced astrogliosis.

Our transcriptomic analyses demonstrated a dual astrocytic response depending on time post-injury and lesion severity. Following moderate SCI, astrocytes displayed a protective role and showed no changes (1 week) and even down-regulated (2 weeks) expression of transcripts involved in immune response. On the other hand, astrocytes response after severe SCI seems to be detrimental by an upsurge expression of inflammatory genes (1 week) and prevention of extracellular re-modeling (2 weeks) (3). These are the first concrete evidence of a heterogeneous astrocytic response that is driven not only by lesion severity but also time after injury (Figure 1).

**Figure 1:**
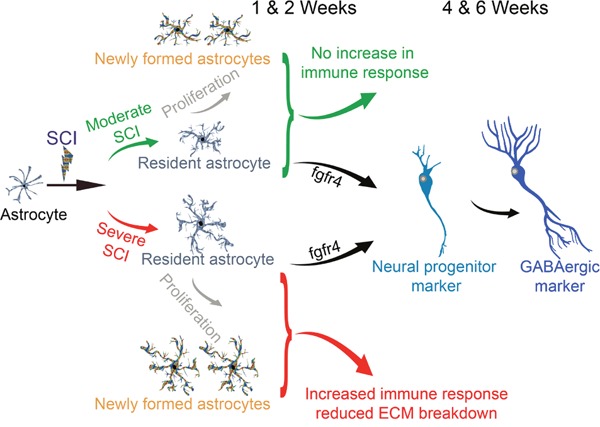
Schematic cartoon displaying summary of astrocytic responses following SCI

In parallel, using pathway analyses, we also identified in astrocytes the induction of the neural stem cell lineage and the over-expression of the neuronal progenitor gene βIII-tubulin (*Tubb3*, also known as *TUJ1*). We confirmed βIII-tubulin protein expression at tissue level using immunohistochemistry and at single cell level using FACS analyses. The sub-population of astrocytes that express βIII-tubulin was only found within 750μm distance to the lesion epicenter. Astrocytes co-expressing βIII-tubulin, also displayed alterations in their morphology from typical stellate shape to classical neuronal progenitor cells with bipolar or multipolar processes. Given that SCI induces astrocytic proliferation, we injected BrdU in Aldh1l1-EGFP mice after injury to determine the origin of eGFP/βIII-tubulin co-expressing cells. BrdU incorporation was observed into newly formed astrocytes but not in eGFP/βIII-tubulin-expressing astrocytes. This suggests that it is the resident mature astrocytes, rather than newly formed astrocytes, that undergo transdifferentiation towards neuronal lineage (Figure 1). Time-dependent analyses revealed that astrocytic conversion towards neuronal lineage starts as early as 72 hours, peaking between 1-2 weeks and continues to a lower degree up to 6 weeks after both moderate and severe SCI. Further immunostaining, using mature neuronal markers, showed that transdifferentiating astrocytes eventually express GABAergic, but not glutamatergic, markers. Moreover, we identified the fibroblast growth factor receptor 4 (Fgfr4) as a potential player responsible for SCI-induced astrocytic transdifferentiation towards neuronal lineage. Fgfr4 indeed promotes embryonic stem cell differentiation towards neuronal lineage [4] and showed pronounced over-expression from 72 hours following lesion at both RNA and protein level.

Although other recent studies had shown limited astrocytes conversion towards neuronal lineage upon enforced expression of neurogenic factors, none had reported a spontaneous injury-induced astroglial transdifferentiation *in vivo* [5-8]. Our results show, that following SCI, resident astrocytes have an *intrinsic* capacity to undergo transdifferentiation towards neuronal lineage. Further studies aimed at stimulating this intrinsic pathway in astrocytes to convert a larger population towards neuronal phenotype may provide a new therapeutic strategy to replace demised neurons and improve functional outcomes after SCI. Our on-going work involves in depth investigation of molecular pathways involved in this intrinsic injury-induced astrocytic conversion towards neuronal linage as well as its functional role in SCI pathophysiology.

## References

[1] Karimi-Abdolrezaee S, Billakanti R (2012). Molecular neurobiology.

[2] Sofroniew MV (2009). Trends Neurosci.

[3] Noristani HN, Sabourin JC, Boukhaddaoui H, Chan-Seng E, Gerber YN, Perrin FE (2016). Molecular neurodegeneration.

[4] Kunath T, Saba-El-Leil MK, Almousailleakh M, Wray J, Meloche S, Smith A (2007). Development.

[5] Guo Z, Zhang L, Wu Z, Chen Y, Wang F, Chen G (2014). Cell Stem Cell.

[6] Magnusson JP, Goritz C, Tatarishvili J, Dias DO, Smith EM (2014). Science.

[7] Sirko S, Behrendt G, Johansson PA, Tripathi P, Costa M (2013). Cell Stem Cell.

[8] Su Z, Niu W, Liu ML, Zou Y, Zhang CL (2014). Nature communications.

